# Storage Temperature Effects on *Bacillus* Spores and *Lactobacillus acidophilus* Viability

**DOI:** 10.1155/ijfo/3966944

**Published:** 2025-09-22

**Authors:** Jessie Payne, Danielle Bellmer, Ravi Jadeja, Brooke Holt, Bailey Holcomb, Sarah Spring

**Affiliations:** ^1^ Department of Animal and Food Science, Oklahoma State University, Stillwater, Oklahoma, USA, okstate.edu; ^2^ Robert M. Kerr Food and Agricultural Products Center, Oklahoma State University, Stillwater, Oklahoma, USA, okstate.edu; ^3^ Department of Biosystems and Agricultural Engineering, Oklahoma State University, Stillwater, Oklahoma, USA, okstate.edu; ^4^ Department of Horticulture and Landscape Architecture, Oklahoma State University, Stillwater, Oklahoma, USA, okstate.edu

**Keywords:** *Bacillus subtilis*, food processing, *Lactobacillus*, probiotics, storage stability

## Abstract

This study investigated the impact of various storage temperatures on the viability of four commercial probiotic strains: *Lactobacillus acidophilus* (*LA-1*) vegetative cells, *Bacillus subtilis* 1 spores, *ProSilience Bacillus subtilis HU58* (*HU58*) spores, and *Bacillus coagulans GBI-30*, *6086* (*BC30*) spores. These probiotics were incorporated into cookies and crackers, which were then stored at 25°C, 4°C, and −18°C for 12 months. Evaluations were conducted at eight different time points throughout the storage period. Among the probiotics tested, the *B. subtilis* spores exhibited the greatest stability, showing < 2 log reductions under all conditions over the 12 months. In contrast, *LA-1* cells were the least stable, falling below the minimum therapeutic level for probiotic microorganisms in a food product (10^6^ CFU/g) after just 2 months in crackers and 4 months in cookies. *BC30* spores were more sensitive to temperature changes than the other *Bacillus* strains (*B. subtilis 1* and *HU58*), with > 4 log reductions. This study also revealed that different probiotics have distinct optimal storage conditions. However, storage temperature had no significant effect on the viability of *B. subtilis* 1 spores, *BC30* spores, and *LA-1* vegetative cells. In contrast, *HU58* spores were notably affected by temperature during the final months of storage. Specifically, samples held at 25°C showed significantly higher log reductions compared to those stored at cooler temperatures, highlighting HU58’s sensitivity to temperature, particularly with longer storage periods. Throughout the storage period, both *BC30* spores and *LA-1* cells experienced substantial increases in log reductions. Overall, this study highlights the importance of selecting appropriate storage conditions for different probiotic strains to maintain their viability in food products over extended periods.

## 1. Introduction

The concept of probiotics dates back to the early 1900s, when Elie Metchnikoff hypothesized that the longevity of Bulgarian peasants was linked to their consumption of fermented milk containing beneficial bacteria [1]. Today, the World Health Organization defines probiotics as “live microorganisms which, when administered in adequate amounts, confer a health benefit on the host” [2]. Common probiotic genera include *Lactobacillus* and *Bifidobacterium*, with species such as *L. acidophilus*, *L. paracasei*, *B. bifidum*, and *B. adolescentis* playing key roles in gut health. However, the viability of these vegetative probiotics is often compromised by factors such as temperature, oxidation, and storage duration [3].

To overcome these limitations, spore‐forming *Bacillus* species have gained interest due to their resilience in harsh environments. *Bacillus* spores can withstand heat, oxidation, and digestive conditions, making them suitable for a broader range of food products. Species like *Bacillus coagulans* and *Bacillus subtilis* have been shown to promote digestive and immune health [4, 5], but only specific strains within this genus offer probiotic benefits [6–8].

Storage temperature plays a critical role in probiotic viability. Vegetative strains are particularly sensitive to elevated temperatures, which can accelerate their decline [9], while most spore‐formers exhibit higher thermal resistance. Water activity also influences stability, with lower water activity increasing thermal resistance [10]. Freezing can preserve viability but only under controlled conditions [11].

Although refrigeration is standard for probiotic storage, some vegetative species remain stable at 25°C in specific matrices. For example, Klu and Chen [12] showed that peanut butter preserved species of *Lactobacillus*, *Lactococcus*, *Bifidobacterium*, and *Streptococcus*—including *L. acidophilus* (CUL 60, CUL 21), *B. bifidum* (CUL 20), *B. lactis* (CUL 34), other species such as *B. breve*, *B. longum*, *L. brevis*, *L. bulgaricus*, *L. casei*, *L. gasseri*, *L. paracasei*, *L. plantarum*, *L. rhamnosus*, *L. salivarius*, *Lactococcus lactis*, and *Streptococcus thermophilus*—with minimal viability loss over 48–52 weeks.

Most research has focused on vegetative strains, while data on *Bacillus* spore stability under different storage conditions remain limited. Studies on *B. coagulans* strains (e.g., MTCC 5856 and GBI‐30, 6086) show that −18°C storage best preserves viability across various food products, while ambient storage leads to significant loss within days [13, 14]. Given the promising potential of probiotics in food products, it is crucial to explore how these strains, particularly spores, behave under storage conditions relevant to different food matrices, including bakery products.

The addition of probiotic bacteria to bakery products is a promising approach to enhance the functional value of commonly consumed foods. Probiotics, such as *Lactobacillus* and *Bacillus*, have been shown to offer numerous health benefits, including improving gut health, enhancing immune function, and potentially reducing the risk of certain gastrointestinal disorders. Incorporating probiotics into bakery products, which are widely consumed daily, provides an effective means of delivering these health benefits in a convenient and palatable form. Moreover, the ability to maintain the viability of probiotics throughout the baking process and during storage is essential for ensuring their efficacy. By integrating probiotics into dough, bakery products can become a more accessible source of functional ingredients, potentially contributing to the growing demand for foods that support overall health and well‐being. This study addresses the knowledge gap by evaluating the long‐term viability of *Bacillus* spores in baked products with medium‐ and low‐water activity under different temperature conditions. We aim to determine how storage temperature influences the stability of these strains over time and identify when viability drops below 10^6^ CFU/g, a threshold below which health benefits may no longer be effective. This innovative approach also aligns with the increasing consumer preference for functional foods that combine convenience, taste, and health‐promoting properties.

## 2. Materials and Methods

The current manuscript shares similarities with the article “Impact of Relative Humidity on *Bacillus* Probiotic Viability During Storage in Baked Products” in that both studies employed comparable methodologies. In particular, the baking, inoculation, and enumeration procedures were similar [15]. At the same time, the key difference lies in the storage conditions investigated—temperature effects in the present study versus relative humidity effects in the cited work. Together, these approaches provide complementary insights into factors that influence probiotic stability in baked products.

### 2.1. Probiotic Bacteria

For this study, four probiotic strains were acquired. Three of these strains were provided by commercial manufacturers in the United States. Strain selection was based on the probiotic manufacturing companies that contributed strains for research purposes. As requested by the manufacturing companies, not all probiotic strain numbers and names will be listed in this manuscript. All probiotics used are generally recognized as safe (GRAS). *B. subtilis 1* spores [16] and *Bacillus coagulans GBI-30, 6086* (*BC30*) spores [17] have GRAS notices from the US Food and Drug Administration (FDA), while *Lactobacillus acidophilus* (*LA-1*) vegetative cells and *ProSilience Bacillus subtilis HU58™* (*HU58*) spores have GRAS notices from independent expert panels (*LA-1*: Honeycombs Industries; *HU58*: Soni & Associates Inc., 2012). The *L. acidophilus* strain used in this study is catalogued internally under the designation *LA-1*, following institutional and commercial confidentiality policies. Details on concentration, culture media, and incubation conditions are provided in Table 1. Strains were donated in spray‐dried form, so no regrowth inoculum was needed. The spray drying process is proprietary information kept by the manufacturing companies.

**Table 1:** Probiotic strains and their enumeration conditions.

**Strain**	**Manufacturer concentration**	**Culture media**	**Incubation conditions**
*Bacillus subtilis* 1 spores	3.0 × 10^11^ CFU/g	Fisher Scientific Tryptic soy agar (TSA)	37^°^C + /−2^°^C 24 h
ProSilience *Bacillus subtilis* HU58 spores	1.5 × 10^10^ CFU/g	Fisher Scientific Tryptic soy agar (TSA)	37^°^C + /−2^°^C 24 h
*Bacillus coagulans* GBI‐30, 6086 (BC30) spores	1.5 × 10^10^ CFU/g	Fisher Scientific Tryptic soy agar (TSA)	37^°^C + /−2^°^C 48 h
*Lactobacillus acidophilus* (LA‐1) vegetative cells	1.2 × 10^10^ CFU/g	HI Media De Man, Rogosa, and Sharpe agar (MRS)	37^°^C + /−2^°^C 48 h

It should be noted that *LA-1* vegetative cells were used as a comparative baseline (heat‐sensitive control) for evaluating the *Bacillus* spores due to their status as one of the most widely used probiotics on the market. This study is aimed at determining whether *B. coagulans* and *B. subtilis* spores are viable alternatives to the heat‐sensitive *LA-1* cells.

### 2.2. Food Products

Cookies and crackers were selected as carriers for probiotics in this study due to their medium (cookies) and low (crackers) water activity levels and their requirement for a baking process. This approach allows for the differentiation between vegetative cells (*LA-1*) and spore bacteria (*BC30*, *B. subtilis 1*, and *HU58*) by subjecting them to a heat process under different water activity conditions. Cookies were baked following the American Association of Cereal Chemists (AACC) International method 10‐54.01, which details the baking quality of cookie flour using a micro wire‐cut formulation [18]. The cookie dough was prepared with the following ingredients: 80.0‐g all‐purpose flour, 17.6‐g deionized water, 33.6‐g sucrose, 0.8‐g nonfat dry milk, 1.0‐g sodium chloride, 0.8‐g sodium bicarbonate, 32.0‐g shortening, 1.2‐g corn syrup, and 0.4‐g ammonium bicarbonate [18].

Crackers were baked according to a modified version of the US Department of Agriculture (USDA) baking procedure [19]. The cracker dough was prepared with the following ingredients: 75.0‐g all‐purpose flour, 39.45‐g distilled water, 6.25‐g sucrose, 25.65‐g shortening, and 1.50‐g sodium chloride. The baking procedure for both products was conducted in a Frigidaire 30‐in. 4‐Element 5.3‐cu ft Freestanding Electric Range oven (Frigidaire, Charlotte, North Carolina, United States).

#### 2.2.1. Inoculation of Baked Products With Probiotics

Cracker and cookie doughs were prepared following established protocols. A 2% concentration (*w*/*w*) of spray‐dried probiotic culture was added to the flour before dough mixing. Probiotics were incorporated using a KitchenAid mixer for cookie dough and a food processor for cracker dough. To assess the uniform distribution of probiotics, homogeneity tests were conducted using food dye. As an additional verification method, the dye was mixed with a powdered component before incorporation into the dough; during testing, the dyed powder was fully incorporated, indicating effective homogenization. In addition, during preliminary trials, a test batch of each dough type was analyzed at multiple locations within the dough matrix to confirm uniform probiotic distribution. Following mixing, the doughs were portioned and baked at 400°F (204°C) for 10 min. Samples of unbaked dough from each batch were retained for enumeration of initial probiotic counts.

### 2.3. Enumeration of Probiotic Strains

Before the study, initial probiotic concentration levels were verified and compared with those reported by the manufacturers. The initial CFU/g concentrations of probiotics in the dough before baking were established and used as initial concentration values.

Samples stored under each temperature condition were analyzed at eight intervals over 12 months. Before enumeration, samples were equilibrated to 25°C (approximately 30 min). Subsequently, 5.0 g of the product (cookie or cracker) was weighed and placed into a sterile stomacher bag. The enumeration methods, which vary by probiotic, are detailed in Sections 2.3.1 and 2.3.2. Additionally, these enumeration methods were determined by the providing manufacturing companies.

#### 2.3.1. *B. subtilis* 1 Spores, *B. coagulans* Spores, and *LA-1* Vegetative Cells

Samples containing *B. subtilis 1* spores, *BC30* spores, and *LA-1* cells were stomached with 95 mL of 0.1% sterile buffered peptone water for 60 seconds using an EasyMix Blender (Biomerieux, France). Subsequently, 10 mL of the stomached sample was transferred to a sterile test tube. Samples of *B. subtilis* 1 and *BC30* spores were subjected to heat shocking at 68^°^C + /−2^°^C for 20 min, following the protocol of Jafari et al. [20], using a Cole‐Parmer StableTemp Water Bath (Cole‐Parmer, Illinois, United States). The purpose of heat shocking the *Bacillus* samples was to ensure that the enumerated bacteria were *Bacillus* spores rather than vegetative cells or other microbes. In contrast, *LA-*1 samples were not subjected to heat shock due to significant reductions in vegetative cell counts reported by De Angelis et al. [21]. Following heat shock, the samples were diluted in sterile, buffered peptone water and plated using the pour‐plate method. The media and incubation conditions for each probiotic are specified in Table 1.

#### 2.3.2. HU58

Samples containing *HU58* spores were stomached with 95 mL of 0.1 M phosphate‐buffered saline (PBS) for 60 seconds using an EasyMix Blender (Biomerieux, France). Subsequently, 10 mL of the stomached sample was transferred to a sterile test tube. The samples were then heat‐shocked at 80^°^C + /−2^°^C for 10 min using a Cole‐Parmer StableTemp Water Bath (Cole‐Parmer, Illinois, United States), following the enumeration protocol provided by the manufacturer. *HU58* samples were further diluted in 0.1 M PBS and plated using the spread plate method. Incubation conditions for *HU58* spores are detailed in Table 1.

### 2.4. Temperature Storage Conditions

To evaluate the viability of *Bacillus* as a probiotic under different storage temperatures, samples were kept at three conditions: room temperature (25°C), refrigeration (4°C), and freezing (−18°C). The samples were prepared and inoculated following established protocols and then stored in Ziplock bags at their respective temperatures. Ziplock bags were selected for their convenience in sample organization and accessibility while ensuring exposure to the designated temperatures.

The polyethylene‐based Ziplock bags used have a vapor permeability of approximately 300 mL/100 in.^2^ for oxygen and 1.0 g/100 in.^2^ for water [22]. This level of permeability means that the samples are susceptible to the effects of environmental conditions, including temperature fluctuations, as both oxygen and moisture can permeate through the bags. Thus, the samples’ stability may be influenced by the temperature at which they are stored and by the interaction of these environmental factors over time.

To monitor the impact of these conditions, probiotic enumeration was conducted at eight intervals over 12 months: 0 days and 1, 2, 4, 6, 8, 10, and 12 months. This approach allowed for the assessment of how storage temperature and environmental factors affect the viability of the *Bacillus* samples over time.

### 2.5. Water Activity

The water activity of the cookies and crackers was measured using a Neutec Group Inc. LabSwift‐a_w_ water activity meter (Novasina AG, Lachen, Switzerland). Two samples from each baked product, temperature condition, and time point were assessed for their water activity, with the value averaged (*n* = 48 for each baked product and temperature condition). This procedure was replicated across three independent experiments to determine the average water activity and standard deviation for each type of baked product.

### 2.6. Statistical Analysis

Log reductions (log(*N*_0_/*N*)) were calculated based on the logarithmic difference in the initial dough count before baking (*N*_0_) and the final count after each experiment (*N*). Log reduction at 0 days is the difference between the initial counts before baking and the final counts after the baking process. At Day 0, log reductions reflect the impact of the baking process, with reductions attributed to the fact that only spores and a few vegetative cells survive baking.

Statistical analysis was performed using ANOVA, and mean differences were assessed with Tukey’s HSD test at the significance level of *p* < 0.05. All analyses were carried out using SAS Software, Version 3.81 (Enterprise Edition, SAS Institute Inc., Cary, North Carolina, United States). The experiments were replicated three times independently.

## 3. Results

Each probiotic strain (spore [*BC30*, *HU58*, and *B. subtilis* 1] or vegetative cell [*LA-1*]) had a specific starting concentration. *B. subtilis 1* spores had the highest initial concentration, with 11.54 log_10_/g for cookies and 10.86 log_10_/g for crackers. *B. subtilis HU58* spores, *BC30* spores, and *LA-1* vegetative cells had similar starting concentrations: *HU58* started at 10.20 log_10_/g for cookies and 10.02 log_10_/g for crackers; *BC30* began at 9.88 log_10_/g for cookies and 9.98 log_10_/g for crackers; *LA-1* started at 9.56 log_10_/g for cookies and 10.00 log_10_/g for crackers on average.

In the current study, the impact of baking on probiotic viability is illustrated at Day 0, showing the average difference between the initial concentration in cookie dough and the concentration in the baked product. *B. subtilis 1* experienced a reduction of 0.5 logs in cookies and 1.2 logs in crackers. *HU58* was reduced by 0.7 logs in cookies and 1.0 logs in crackers. *BC30* and *LA-1* showed the highest reductions due to baking. *BC30* decreased by 2.3 logs in cookies and 1.9 logs in crackers, while *LA-1* decreased by 3.4 logs in cookies and 3.8 logs in crackers. Overall, baking had a more detrimental effect on probiotics in crackers compared to cookies, likely due to the crackers’ lower surface area (See Tables 2‐9).

**Table 2:** Effect of storage temperature on the log reductions (log (*N*_0_/*N*)) of *B. subtilis* 1 spores in cookies.

	**Freezer temperature (**−**18°C)**	**Refrigerator temperature (4°C)**	**Room temperature (25°C)**
	**Log reductions (**log**N**_0_/**N** **)**		
Day 0	0.5 ± 0.1 Aa	0.5 ± 0.1 Aa	0.5 ± 0.1 Aa
1 month	1.0 ± 0.1 Aa	1.2 ± 0.3 Aa	1.1 ± 0.2 Aa
2 months	1.0 ± 0.1 Aa	1.2 ± 0.1 Aa	1.1 ± 0.2 Aa
4 months	1.0 ± 0.1 Aa	1.1 ± 0.2 Aa	1.2 ± 0.1 Aa
6 months	1.1 ± 0.1 Aa	1.1 ± 0.1 Aa	1.2 ± 0.2 Aa
8 months	1.0 ± 0.1 Aa	1.1 ± 0.2 Aa	1.1 ± 0.2 Aa
10 months	1.1 ± 0.1 Aa	1.2 ± 0.1 Aa	1.3 ± 0.1 Aa
12 months	1.0 ± 0.0 Aa	1.1 ± 0.1 Aa	1.2 ± 0.2 Aa

*Note:* *N*_0_ is the initial dough count before baking, and *N* is the final count after each experiment. Different capital letters (A) in the same row denote significant differences (*p* ≤ 0.05) in log reductions between the different holding temperatures. Different lowercase letters (a) in the same column denote significant differences (*p* ≤ 0.05) in log reductions between the same holding temperature over the storage period.

**Table 3:** Effect of storage temperature on the log reductions (log (*N*_0_/*N*)) of *B. subtilis HU58* spores in cookies.

	**Freezer temperature (**−**18°C)**	**Refrigerator temperature (4°C)**	**Room temperature (25°C)**
	**Log reductions (**log**N**_0_/**N** **)**		
Day 0	0.7 ± 0.7 Aa	0.7 ± 0.7 Aa	0.7 ± 0.7 Aa
1 month	0.5 ± 0.1 Aa	0.4 ± 0.2 Aa	0.5 ± 0.1 Aa
2 months	0.4 ± 0.4 Aa	0.4 ± 0.1 Aa	0.5 ± 0.2 Aa
4 months	0.4 ± 0.4 Aa	0.4 ± 0.2 Aa	0.5 ± 0.4 Aa
6 months	0.4 ± 0.4 Aa	0.4 ± 0.3 Aa	0.6 ± 0.0 Aa
8 months	0.4 ± 0.1 Ba	0.5 ± 0.1 ABa	0.6 ± 0.1 Aa
10 months	0.5 ± 0.1 Ba	0.6 ± 0.2 ABa	0.8 ± 0.1 Aa
12 months	0.6 ± 0.3 Ba	0.7 ± 0.2 ABa	0.8 ± 0.1 Aa

*Note:* *N*_0_ is the initial dough count before baking, and *N* is the final count after each experiment. Different capital letters (A) in the same row denote significant differences (*p* ≤ 0.05) in log reductions between the different holding temperatures. Different lowercase letters (a) in the same column denote significant differences (*p* ≤ 0.05) in log reductions between the same holding temperature over the storage period.

**Table 4:** Effect of storage temperature on the log reductions (log (*N*_0_/*N*)) of *B. coagulans BC30* spores in cookies.

	**Freezer temperature (**−**18°C)**	**Refrigerator temperature (4°C)**	**Room temperature (25°C)**
	**Log reductions (**log**N**_0_/**N** **)**		
Day 0	2.3 ± 0.3 Ab	2.3 ± 0.3 Ac	2.3 ± 0.3 Ac
1 month	2.5 ± 0.2 Ab	2.6 ± 0.6 Abc	2.5 ± 0.4 Ac
2 months	2.6 ± 0.0 Ab	2.6 ± 0.3 Abc	2.6 ± 0.1 Abc
4 months	2.5 ± 0.5 Ab	2.6 ± 0.4 Abc	2.6 ± 0.3 Abc
6 months	2.5 ± 0.1 Ab	2.7 ± 0.2 Abc	2.7 ± 0.3 Abc
8 months	3.4 ± 0.2 Aab	3.6 ± 0.1 Aab	3.6 ± 0.2 Aab
10 months	4.0 ± 0.9 Aa∗	3.9 ± 0.6 Aa∗	4.1 ± 0.7 Aa∗
12 months	4.0 ± 0.4 Aa∗	4.0 ± 0.6 Aa∗	4.4 ± 0.4 Aa∗

*Note:* *N*_0_ is the initial dough count before baking, and *N* is the final count after each experiment. Different capital letters (A) in the same row denote significant differences (*p* ≤ 0.05) in log reductions between the different holding temperatures. Different lowercase letters (a) in the same column denote significant differences (*p* ≤ 0.05) in log reductions between the same holding temperature over the storage period. Values with an asterisk (∗) indicate below the required viability level (6 log CFU).

**Table 5:** Effect of storage temperature on the log reductions (log (*N*_0_/*N*)) of *L. acidophilus LA-1* vegetative cells in cookies.

	**Freezer temperature (**−**18°C)**	**Refrigerator temperature (4°C)**	**Room temperature (25°C)**
	**Log reductions (**log (**N**_0_/**N**)**)**		
Day 0	3.4 ± 0.6 Ac	3.4 ± 0.6 Aa	3.4 ± 0.6 Aa
1 month	3.4 ± 0.8 Abc	3.5 ± 0.3 Aa	3.5 ± 0.3 Aa
2 months	3.5 ± 0.2 Aabc	3.9 ± 0.0 Aa	3.6 ± 0.6 Aa
4 months	3.5 ± 0.3 Aabc	4.0 ± 0.7 Aa	3.7 ± 0.3 Aa
6 months	4.4 ± 0.4 Aabc∗	4.1 ± 0.7 Aa∗	3.9 ± 0.2 Aa∗
8 months	4.2 ± 0.3 Aabc∗	4.2 ± 0.1 Aa∗	4.1 ± 0.3 Aa∗
10 months	4.7 ± 0.3 Aab∗	4.2 ± 0.3 Aa∗	4.2 ± 0.5 Aa∗
12 months	4.7 ± 0.4 Aa∗	4.5 ± 0.5 Aa∗	4.6 ± 0.6 Aa∗

*Note:* *N*_0_ is the initial dough count before baking, and *N* is the final count after each experiment. Different capital letters (A) in the same row denote significant differences (*p* ≤ 0.05) in log reductions between the different holding temperatures. Different lowercase letters (a) in the same column denote significant differences (*p* ≤ 0.05) in log reductions between the same holding temperature over the storage period. Values with an asterisk (∗) indicate below the required viability level (6 log CFU).

**Table 6:** Effect of storage temperature on the log reductions (log (*N*_0_/*N*)) of *B. subtilis* 1 spores in crackers.

	**Freezer temperature (**−**18°C)**	**Refrigerator temperature (4°C)**	**Room temperature (25°C)**
	**Log reductions (**log (**N**_0_/**N**)**)**		
Day 0	1.2 ± 0.1 Aa	1.2 ± 0.1 Aa	1.2 ± 0.1 Aa
1 month	1.3 ± 0.5 Aa	1.2 ± 0.6 Aa	1.5 ± 0.3 Aa
2 months	1.2 ± 0.2 Aa	1.3 ± 0.2 Aa	1.4 ± 0.2 Aa
4 months	1.3 ± 0.4 Aa	1.2 ± 0.1 Aa	1.5 ± 0.1 Aa
6 months	1.1 ± 0.1 Aa	1.3 ± 0.0 Aa	1.6 ± 0.5 Aa
8 months	1.5 ± 0.2 Aa	1.4 ± 0.0 Aa	1.6 ± 0.1 Aa
10 months	1.4 ± 0.1 Aa	1.6 ± 0.4 Aa	1.8 ± 0.5 Aa
12 months	1.6 ± 0.4 Aa	1.6 ± 0.0 Aa	1.8 ± 0.2 Aa

*Note:* *N*_0_ is the initial dough count before baking, and *N* is the final count after each experiment. Different capital letters (A) in the same row denote significant differences (*p* ≤ 0.05) in log reductions between the different holding temperatures. Different lowercase letters (a) in the same column denote significant differences (*p* ≤ 0.05) in log reductions between the same holding temperature over the storage period.

**Table 7:** Effect of storage temperature on the log reductions (log (*N*_0_/*N*)) of *B. subtilis HU58* spores in crackers.

	**Freezer temperature (**−**18°C)**	**Refrigerator temperature (4°C)**	**Room temperature (25°C)**
	**Log reductions (**log (**N**_0_/**N**)**)**		
Day 0	1.0 ± 0.5 Aa	1.0 ± 0.5 Aa	1.0 ± 0.5 Aa
1 month	1.2 ± 0.3 Aa	1.1 ± 0.4 Aa	1.1 ± 0.2 Aa
2 months	1.3 ± 0.1 Aa	1.2 ± 0.4 Aa	1.1 ± 0.2 Aa
4 months	1.3 ± 0.2 Aa	1.4 ± 0.2 Aa	1.3 ± 0.2 Aa
6 months	1.2 ± 0.1 Aa	1.4 ± 0.2 Aa	1.3 ± 0.3 Aa
8 months	1.4 ± 0.5 Aa	1.3 ± 0.2 Aa	1.5 ± 0.2 Aa
10 months	1.4 ± 0.3 Aa	1.5 ± 0.3 Aa	1.6 ± 0.1 Aa
12 months	1.4 ± 0.3 Aa	1.5 ± 0.3 Aa	1.7 ± 0.1 Aa

*Note:* *N*_0_ is the initial dough count before baking, and *N* is the final count after each experiment. Different capital letters (A) in the same row denote significant differences (*p* ≤ 0.05) in log reductions between the different holding temperatures. Different lowercase letters (a) in the same column denote significant differences (*p* ≤ 0.05) in log reductions between the same holding temperature over the storage period.

**Table 8:** Effect of storage temperature on the log reductions log (*N*_0_/*N*) of *B. coagulans BC30* spores in crackers.

	**Freezer temperature (**−**18°C)**	**Refrigerator temperature (4°C)**	**Room temperature (25°C)**
	**Log reductions (**log (**N**_0_/**N**)**)**		
Day 0	1.9 ± 0.2 Ac	1.9 ± 0.2 Ac	1.9 ± 0.2 Ac
1 month	2.2 ± 0.7 Ac	2.4 ± 0.8 Abc	2.5 ± 0.8 Abc
2 months	2.6 ± 0.2 Abc	2.3 ± 0.3 Abc	2.3 ± 0.6 Abc
4 months	2.3 ± 0.1 Ac	2.2 ± 0.1 Abc	2.2 ± 0.2 Abc
6 months	2.6 ± 0.7 Abc	2.3 ± 0.2 Abc	2.2 ± 0.5 Abc
8 months	3.0 ± 0.4 Aab	3.4 ± 0.1 Aab	3.4 ± 0.2 Aab
10 months	3.8 ± 0.8 Aab∗	4.2 ± 0.7 Aa∗	4.2 ± 0.6 Aa∗
12 months	4.4 ± 0.6 Aa∗	4.5 ± 0.8 Aa∗	4.5 ± 0.8 Aa∗

*Note:* *N*_0_ is the initial dough count before baking, and *N* is the final count after each experiment. Different capital letters (A) in the same row denote significant differences (*p* ≤ 0.05) in log reductions between the different holding temperatures. Different lowercase letters (a) in the same column denote significant differences (*p* ≤ 0.05) in log reductions between the same holding temperature over the storage period. Values with an asterisk (∗) indicate below the required viability level (6 log CFU).

**Table 9:** Effect of storage temperature on the log reductions (log (*N*_0_/*N*)) of *L. acidophilus* (*LA-1*) vegetative cells in crackers.

	**Freezer temperature (**−**18°C)**	**Refrigerator temperature (4°C)**	**Room temperature (25°C)**
	**Log reductions (**log (**N**_0_/**N**)**)**		
Day 0	3.8 ± 0.5 Abc	3.8 ± 0.5 Ab	3.8 ± 0.5 Ab
1 month	3.6 ± 0.4 Ac	3.6 ± 0.2 Ab	3.8 ± 0.0 Ab
2 months	4.2 ± 0.4 Aabc∗	4.2 ± 0.0 Aab∗	4.5 ± 0.1 Aab∗
4 months	4.2 ± 0.4 Aabc∗	4.3 ± 0.5 Aab∗	4.1 ± 0.4 Aab∗
6 months	4.7 ± 0.2 Aabc∗	4.4 ± 0.3 Aab∗	4.5 ± 0.2 Aab∗
8 months	4.9 ± 0.7 Aab∗	4.6 ± 0.5 Aab∗	4.5 ± 0.6 Aab∗
10 months	5.2 ± 0.5 Aa∗	4.8 ± 0.7 Aab∗	4.9 ± 0.8 Aab∗
12 months	5.4 ± 0.4 Aa∗	5.2 ± 0.3 Aa∗	5.1 ± 0.3 Aa∗

*Note:* *N*_0_ is the initial dough count before baking, and *N* is the final count after each experiment. Different capital letters (A) in the same row denote significant differences (*p* ≤ 0.05) in log reductions between the different holding temperatures. Different lowercase letters (a) in the same column denote significant differences (*p* ≤ 0.05) in log reductions between the same holding temperature over the storage period. Value with asterisk (∗) indicate below the required viability level (6 log CFU).

### 3.1. Effect of Storage Temperature—Cookies

Two food matrices, cookies and crackers, were used to evaluate the impact that storage temperature could have on the viability of *Bacillus* spores. At Day 0, the effect of baking was assessed by comparing the log reduction between the initial counts before the baking process (*N*_0_) and the counts after baking (*N*). For *B. subtilis* 1 spores in cookies, storage temperature had no significant effect on viability over the 12 months of storage (Table 2). Although 25°C storage tended to result in higher log reductions compared to 4°C and −18°C, these differences were not statistically significant. Furthermore, no significant increase in log reductions was observed over the 12‐month storage period at any temperature for this probiotic (Table 2, nonsignificant [NS]).

Cookies containing *HU58* spores exhibited the most pronounced log reductions when stored at 25°C. This effect was particularly evident at 8, 10, and 12 months, where cookies stored at 25°C showed significantly higher log reductions compared to those stored at −18°C (Table 3, *p* ≤ 0.05). Like *B. subtilis* 1, no substantial changes in log reductions were observed over the 12‐month storage period for any of the tested temperatures (Table 3, NS). Overall, the viability of cookies inoculated with *HU58* was more adversely affected by 25°C storage compared to −18°C conditions.

Samples containing *BC30* displayed similar trends to the other *Bacillus* probiotics concerning the impact of storage temperature. No significant differences were observed between the temperature conditions for *BC30*. However, samples stored at 25°C exhibited higher log reductions compared to those stored at −18°C or 4°C (Table 4, NS). Unlike the two *B. subtilis* spores, *BC30* showed significant variations in log reductions over the 12‐month storage period. Notably, there was a substantial increase in log reductions between the 6 and 8‐month time points. For all storage conditions, samples stored for 10 and 12 months had significantly higher log reductions than those stored at all other time points, except 8 months. Specifically, for 4°C samples, those stored for 8 months had statistically higher log reductions compared to Day 0. Additionally, samples held at 25°C for 8 months exhibited significantly higher log reductions compared to those stored for 0, 1, 2, and 4 months.

Storage temperature did not significantly affect the viability of *LA-1*, with all three temperatures showing statistically similar log reductions at each time point. However, the samples stored at −18°C tended to have higher log reductions (Table 5). Over the 12 months, no significant increases in log reductions were observed for samples stored at 4°C or at 25°C. In contrast, samples stored at −18°C showed a statistically significant increase in log reductions between Day 0, 10 months, and 12 months of storage.

Comparative analysis of the probiotics revealed that the *B. subtilis* spores (*B. subtilis 1* and *HU58*) demonstrated the greatest stability during storage (see Figures S1 and S2, *p* ≤ 0.05). Over the 12‐month storage period, *LA-1* vegetative cells and *BC30* spores exhibited significantly higher log reductions than *B. subtilis 1* and *HU58*. The viable cells of *LA-1* and *BC30* spores showed statistically significant differences at all time points except at 10 and 12 months. At 25°C, *BC30* spores also showed statistical similarity to *LA-1* vegetative cells at the 8‐month time point (see Figures S1 and S2, *p* ≤ 0.05). Across all storage temperatures, the *B. subtilis 1* and *HU58* spores were statistically similar.

### 3.2. Effect of Storage Temperature—Crackers

The second food matrix examined in this study was crackers. For *B. subtilis* 1 spores, the findings were consistent with those observed for cookies. Storage temperature did not significantly affect viability over the 12‐month storage period (Table 6). As with cookies, 25°C storage tended to result in higher log reductions for crackers compared to 4°C and −18°C; however, these differences were not statistically significant. Additionally, no significant differences were observed across any of the storage temperatures throughout the 12 months (Table 6, NS).

Crackers containing *HU58* spores exhibited no significant differences in log reductions across the various storage temperatures at each time point (Table 7, NS). Overall, samples stored at 25°C and at 4°C showed the greatest log reductions, although during the initial months, samples stored at −18°C had higher log reductions. Despite these observations, the differences were not statistically significant. Over the 12‐month storage period, no significant increases in log reductions were observed at any storage temperatures, consistent with the findings for *B. subtilis 1*.

The results for *BC30* spores in both cookie and cracker matrices were consistent, showing no significant differences among the three storage temperatures tested. Overall, samples stored at 25°C and 4°C exhibited the highest log reductions. Notably, there was a substantial increase in log reductions between 6 and 8 months. All storage conditions displayed significant increases in log reductions over the 12 months (Table 8, *p* ≤ 0.05). Specifically, for samples stored at 25°C and 4°C, log reductions at 10 and 12 months were significantly higher compared to all other time points except 8 months. Crackers with *BC30* spores stored for 8 months also had significantly higher log reductions compared to Day 0. Conversely, crackers stored at −18°C for 12 months showed markedly higher log reductions than all other time points. Additionally, crackers analyzed at 8 and 10 months had significantly higher log reductions than those stored for 0, 1, 2, and 4 months.

*LA-1* vegetative cells exhibited the greatest log reductions when stored at −18°C (Table 9). Although samples stored at −18°C showed higher log reductions over the storage period, there were no statistically significant differences among the three storage temperatures. Over the 12 months, log reductions increased significantly for samples stored in both −18°C and 25°C. At −18°C, samples at the 10‐ and 12‐month time points had significantly higher log reductions compared to those at Day 0 and 1 month (Table 9, *p* ≤ 0.05). Similarly, at 25°C, samples analyzed at 12 months had significantly higher log reductions than those at Day 0 and 1 month. Conversely, no significant increases in log reductions were observed for samples stored at 4°C.

When comparing the probiotics, *B. subtilis 1* and *HU58* spores exhibited the lowest log reductions throughout the storage period, indicating the highest stability and consistency with minimal increases in log reductions (see Figures S1 and S2, *p* ≤ 0.05). In both cookies and crackers, *B. subtilis 1* and *HU58* spores remained statistically similar. In contrast, *BC30* spores were statistically different from *B. subtilis* spores, except at Day 0 and 1 month across all temperatures. *LA-1* vegetative cells demonstrated statistically higher log reductions at all time points evaluated, except at 10 and 12 months, where *BC30* spores showed similar log reductions (see Figures S1 and S2, *p* ≤ 0.05). Overall, *B. subtilis 1* and *HU58* spores were the most stable strains compared to the viable cells of *LA-1* and *BC30* spores.

### 3.3. Water Activity

**Figure 1:**
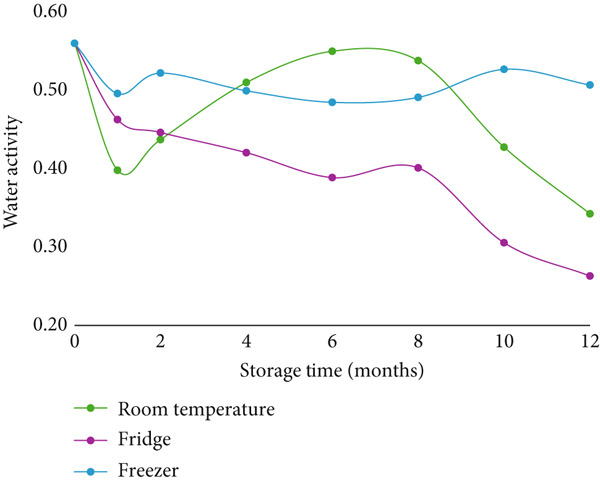
The water activity of cookies during 12 months of storage. Note: Statistical differences are summarized in Table S1.

**Figure 2:**
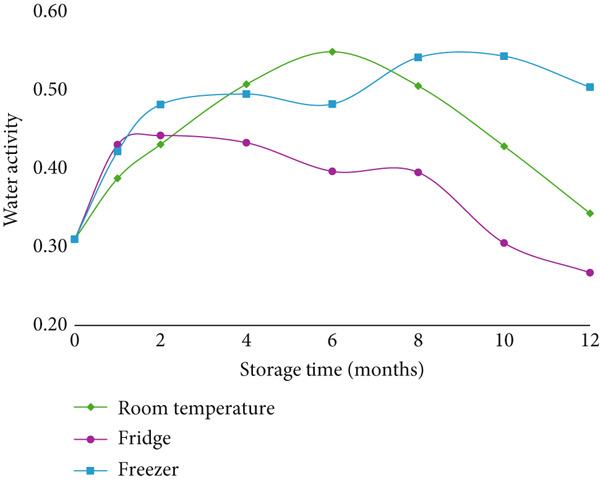
The water activity of crackers during 12 months of storage. Note. Statistical differences are summarized in Table S2.

At each time point, the water activity of the samples was assessed, revealing significant changes in both cookies and crackers over time (Figures 1 and 2). These variations in water activity were attributed to fluctuations in temperature and humidity in the storage environment, as well as the natural migration of moisture from the air into the dry‐baked products. This moisture migration occurs from areas of higher water activity to areas of lower water activity, thereby affecting the texture of the baked goods [23].

The presence of solutes, such as sugar and salt, also influences water activity by binding water molecules. This effect was evident in the samples: crackers, being thin and high in salt, exhibited a lower water activity and a crisp texture, while cookies, which are denser and contain both sugar and salt, had higher moisture content and intermediate water activity [23]. Among the probiotics tested, *B. subtilis HU58* was the only strain to show significant differences in viability under varying temperature conditions (Figures 1 and 2 and Tables S1 and S2). Temperature fluctuations did not notably affect the viability of the other probiotics. Despite the observed changes in water activity, the lack of significant differences in log reductions across temperature conditions suggests that water activity was not a major factor influencing probiotic viability.

*HU58* was the only probiotic to show significant differences in log reductions for cookies stored at −18°C versus at 25°C at the 8‐, 10‐, and 12‐month time points (Table 3). Table 10 shows the effect of water activity on *HU58*. Analysis of water activity over the 12 months revealed significant differences at the 4‐, 10‐, and 12‐month time points (Table 10). Although water activity differed significantly between 4°C and 25°C/−18°C conditions at 4 months, this difference did not correspond to variations in log reductions. At the 8‐month time point, no significant difference in water activity was observed, yet significant differences in log reductions were noted among temperature conditions. Significant differences in both water activity and log reductions were only present at the 10‐ and 12‐month time points (Tables 3 and 10). The lack of consistent correlation between water activity and log reductions suggests that water activity was not a major factor in the observed reductions at the 8‐month time point and may not account for the log reductions at the 10‐ and 12‐month time points either.

**Table 10:** Effect of water activity on the viability of *B. subtilis HU58* spores in cookies.

**Time (months)**	**Freezer temperature (**−**18°C)**	**Refrigerator temperature (4°C)**	**Room temperature (25°C)**
1	0.54 ± 0.0 A	0.63 ± 0.1 A	0.53 ± 0.0 A
2	0.54 ± 0.0 A	0.55 ± 0.1 A	0.59 ± 0.1 A
4	0.57 ± 0.0 A	0.49 ± 0.0 B	0.57 ± 0.0 A
6	0.55 ± 0.0 A	0.46 ± 0.1 A	0.58 ± 0.0 A
8	0.62 ± 0.0 A	0.44 ± 0.1 A	0.49 ± 0.1 A
10	0.58 ± 0.0 A	0.36 ± 0.1 B	0.34 ± 0.0 B
12	0.56 ± 0.0 A	0.31 ± 0.0 B	0.37 ± 0.0 C

*Note:* Different capital letters (A) in the same row denote significant differences (*p* ≤ 0.05) in log reductions between the different holding temperatures.

### 3.4. Interactions Among Variables

Several key variables were evaluated in this study, including water activity, log reductions, storage temperature, storage time, and product type. Examining how these factors interact allowed us to determine which conditions positively or negatively affect probiotic viability (Table 11).

**Table 11:** Multivariate regression summary: Factors affecting log reductions in *Bacillus* probiotics.

**Factor**	***HU58***	***B. subtilis 1***	***BC30***	***LA-1***
Product (cracker vs. cookie)	+0.7038 ∗ ↑	−0.0944 (n.s.)	−0.4820 (n.s.)	−0.3196 (n.s.)
Storage temperature (°C)	+0.0304 ∗ ↑	−0.0101 (n.s.)	+0.0710 (n.s.)	−0.0425 ∗ ↓
Storage time (months)	+0.0337 ∗ ↑	+0.0018 (n.s.)	+0.2360 ∗ ↑	−0.0343 ∗ ↓
Water activity (*a*_w_)	−0.6016 ∗ ↓	−4.8073 (n.s.)	+0.5140 (n.s.)	−0.5151 (n.s.)

*Note:* ↑/↓ = direction of effect on log reduction (positive vs. negative). Significance levels: asterisk (∗) denotes *p* < 0.001 and n.s. denotes not significant (*p* ≥ 0.05).

The multivariate regression analysis demonstrated notable differences in how *HU58*, *B. subtilis 1*, and *BC30* respond to formulation and storage conditions (Table 11). In this analysis, a higher log reduction indicates a greater loss of viable cells and thus reduced probiotic stability, while a lower log reduction reflects improved survival.

*HU58* spores showed the strongest sensitivity to environmental and product variables (Table 11). Cracker formulations led to significantly greater log reductions than cookies (+0.7038), indicating reduced stability in that matrix. Similarly, increasing storage temperature (+0.0304) and longer storage time (+0.0337) both contributed to higher log reductions, suggesting these conditions promote microbial loss. Lower water activity improved *HU58*’s stability, as shown by a significant negative coefficient (−0.6016), meaning the strain was more stable in drier environments.

In contrast, *B. subtilis 1* spores displayed no statistically significant effects from any of the tested factors, although the coefficient for water activity was notably large and negative (−4.8073, n.s.). While not significant, this may indicate a trend toward improved survival at higher moisture levels for this strain, albeit with considerable variability (Table 11).

*BC30* spores exhibited a significant increase in log reduction with longer storage time (+0.2360), indicating a clear decline in probiotic stability over time. Other variables, including product type (−0.4820, n.s.), temperature (+0.0710, n.s.), and water activity (+0.5140, n.s.), were not statistically significant, though the negative coefficient for crackers may suggest a potential protective effect of this matrix (Table 11).

In summary, *HU58* spores were the least stable strain, responding significantly and negatively to multiple environmental factors (Table 11). *B. subtilis 1* spores were more resilient, while *BC30* spores showed a clear vulnerability to extended storage durations. These findings underscore the importance of tailoring processing and packaging strategies to the specific stability profiles of individual probiotic strains.

## 4. Discussion

The objective of this study was to evaluate the long‐term stability of various *Bacillus* spores and *Lactobacillus* vegetative cells in medium and low‐water activity baked products stored under different temperature conditions: room temperature (25°C), refrigeration (4°C), and freezing (−18°C). Probiotic viability is often assessed based on the recommended therapeutic doses, which are strain‐dependent. For this study, a viability level of 6 log CFU/g was selected as the standard, as this is generally considered the minimum effective dose for ensuring the potential health benefits of probiotics in food products. This threshold allows for a consistent benchmark when comparing the stability of different probiotic strains under varying storage conditions.

The probiotic strains evaluated in this study differ significantly in their biological characteristics, particularly in relation to spore formation, thermal tolerance, and mechanisms of probiotic action. LA‐1 is a nonspore‐forming, vegetative probiotic that is highly sensitive to environmental stressors such as heat, oxygen, and low water activity. Its viability is directly linked to its ability to survive and colonize the gut, making it less suitable for applications involving thermal processing. In contrast, the *B. coagulans* (*BC30*) and *B. subtilis* strains (*B. subtilis 1* and *HU58*) are spore‐forming bacteria with robust resistance to heat and desiccation. Their spores can survive harsh processing conditions, including baking, and later germinate in the gastrointestinal tract to exert probiotic effects. Additionally, *Bacillus* spores are known to possess immunomodulatory properties even in their dormant form, offering functional benefits independent of active colonization. These fundamental differences underscore the importance of selecting probiotic strains based on the intended food matrix and processing method. In this study, *LA-1* vegetative cells were included as a widely used, commercially relevant nonspore‐forming probiotic to serve as a benchmark for thermal sensitivity, while *BC30* and both the *B. subtilis* strains were selected for their established spore‐forming capability and known resilience to thermal and environmental stresses. This approach allowed us to comprehensively evaluate probiotic stability across a spectrum of biological characteristics, reflecting real‐world applications in baked food products.

Previous research has shown that the viability of probiotics is influenced by storage temperatures [9, 11–14, 24]. Studies by Simpson et al. [24] and Klu et al. [9] found that higher temperatures, such as 25°C, result in reduced viability of vegetative probiotics (*Bifidobacteria* and *Lactobacilli*). According to Min et al. [3], storing vegetative probiotics (such as *Lactobacillus*) at 25°C exposes them to heat, acid, and oxygen, which can decrease their viability. In contrast, Klu and Chen [12] demonstrated that some probiotics (*Lactobacillus* and *Bifidobacterium*) can remain stable at 25°C, particularly in specific products like peanut butter.

Our findings align with Klu and Chen [12] in that most probiotics maintained their viability across the different storage temperatures, showing no significant losses. However, the *HU58 B. subtilis* strain experienced notable declines in viability at 25°C, consistent with the concerns raised by Min et al. [3] about the impact of ambient conditions on probiotic stability. Overall, while many *Bacillus* spores were stable, there was a trend toward higher log reductions at 25°C, supporting the observations of Klu et al. [9] regarding the detrimental effects of elevated temperatures on probiotic viability.

Although changes in water activity (*a*_w_) were observed over time (as shown in Figures 1 and 2), these fluctuations did not consistently correlate with viability losses. This may be due to the high resistance of *Bacillus* spores to desiccation and osmotic stress, making them less sensitive to changes in water activity compared to vegetative cells. While water activity can influence chemical degradation processes or microbial activity in general, the intrinsic resilience of spores, including thick protective layers and metabolic dormancy, likely reduced the impact of *a*_w_ shifts under the tested conditions. Therefore, despite statistically significant changes in *a*_w_ over time, no direct relationship between *a*_w_ and probiotic viability was evident in most cases. However, interactions between *a*_w_ and temperature—especially at higher temperatures where moisture migration or recrystallization might occur—warrant further study.

Current recommendations suggest storing *Lactobacillus* probiotics at 4°C–5°C to maintain their viability [25]. However, this practice increases transportation and storage costs and raises the risk of probiotic loss if storage conditions fluctuate [3]. The present research found no statistically significant difference in the stability of *B. subtilis* 1 spores, *BC30* spores, and *LA-1* vegetative cells when incorporated into cookies and crackers at 4°C–5°C, 25°C, or −18°C. Therefore, storing these products at 25°C could lower storage costs and mitigate the risk of probiotic loss due to temperature changes.

While cookies containing *HU58* spores did show a significant loss in viability by the end of the 25°C storage period, this strain demonstrated the lowest overall loss compared to other strains across various baking and storage conditions. Therefore, although there was some decline, the loss of *HU58* can be considered minimal. For further reduction in viability loss, 4°C or −18°C could be used. Alternatively, the choice to use *HU58* should be based on the required shelf life of the product.

When probiotics are stored in −18°C conditions, their viability can either be maintained or compromised due to potential damage from ice crystal formation [11]. In the current study, −18°C conditions significantly reduced the viability of *LA-1* due to the absence of a protective coating, which may have led to cellular damage. In contrast, the *Bacillus* spores tested (*B. subtilis 1*, *HU58*, and *BC30*) exhibited optimal viability at −18°C. This finding aligns with Majeed et al. [14], who reported that *B. coagulans* MTCC 5856 maintained its viability at −18°C for up to 12 months.

When comparing the probiotics, *LA-1* consistently showed the highest log reductions over 12 months. After 6 months in cookies and 2 months in crackers, *LA-1* fell below the required viability level of 10^6^ CFU/g. Similarly, *BC30* also dropped below this viability threshold after 8 months of storage in both cookies and crackers. In contrast, both *B. subtilis* strains demonstrated the greatest stability across all tested time and temperature conditions. *B. subtilis* strains experienced < 2 log reductions in both cookies and crackers over 12 months of storage.

To further understand the influence of storage variables on probiotic stability, a multivariate regression analysis was conducted. The results revealed that storage time had a significant positive association with log reduction for both *HU58* and *BC30* (*p* < 0.001), indicating that longer storage durations reduce viability in these strains. Water activity (*a*_w_) was also a significant negative predictor for *HU58* (*p* < 0.001), suggesting reduced stability at higher *a*_w_ levels. Interestingly, *B. subtilis 1* showed a large but NS coefficient for water activity, which may reflect high sensitivity or variability in response to moisture content, though not statistically confirmed. Product type (cracker) significantly impacted *HU58*, resulting in greater log reductions compared to cookies (*p* < 0.001), whereas the effect was not significant for *B. subtilis 1* or *BC30*. Finally, storage temperature showed a significant effect on *HU58*, where higher temperatures led to increased log reductions (*p* < 0.001), supporting the observed trend of reduced probiotic stability at ambient conditions. These findings highlight *HU58*’s sensitivity to multiple environmental variables and underscore the importance of selecting optimal storage conditions based on strain‐specific stability profiles of the probiotics.

## 5. Limitations

In this study, four different probiotic strains from four distinct companies were evaluated for long‐term stability across various storage temperatures. The starting concentrations varied because they were based on what was provided by the respective probiotic manufacturers. Ideally, all probiotics would have been standardized to the same initial CFU/g to enable more direct comparisons. This variation in starting concentrations presents a limitation, as it can affect the magnitude of log reductions and introduce potential bias when comparing strain stability. While the primary objective of this study was to evaluate the relative stability of each strain under different storage conditions, we acknowledge that differences in initial counts complicate cross‐strain comparisons. As such, conclusions regarding comparative performance should be interpreted with caution. Future studies should consider normalizing starting concentrations across strains to strengthen comparative analyses.

Additionally, due to differences in heat shock and enumeration processes among the companies, not all probiotics underwent identical procedures. The enumeration methods used in this research followed the recommendations of the probiotic manufacturers. During storage, cookies and crackers were housed in Ziplock bags, with standard bags used for 25°C and 4°C and freezer‐specific bags for −18°C temperatures. This selection of bags helped maintain the quality of the baked products throughout the 12‐month storage period.

## 6. Conclusion

This study evaluated how different storage conditions influence the long‐term viability of probiotic strains in baked products, specifically cookies and crackers. While *B. subtilis* 1 spores, *BC30* spores, and *LA-1* vegetative cells were generally stable across most temperature conditions, *HU58* spores exhibited a significant decline in viability at 25°C, particularly during the final 2 months of storage. Among all tested strains, *B. subtilis* 1 demonstrated the greatest overall stability, maintaining viability under all temperature conditions, including −18°C, 4°C, and 25°C.

In contrast, *LA-1*, a nonspore‐forming vegetative probiotic, experienced the highest log reductions, especially under frozen conditions, with viability falling below the effective dose threshold within 2–4 months. This highlights the increased vulnerability of vegetative cells to environmental stress and reinforces the importance of selecting strains based on their biological characteristics and intended storage environment.

Multivariate regression analysis provided additional insight into the individual effects of storage variables. Storage time was a significant positive predictor of log reductions for *HU58* and *BC30*, indicating reduced probiotic stability over time. For *HU58*, water activity, higher storage temperature, and product type (cracker) also significantly contributed to increased log reductions. From these findings, there is an emphasis on the need for strain‐specific considerations in probiotic product development. Selecting resilient spore‐forming strains like *B. subtilis* and optimizing formulation and storage conditions can enhance product stability and ensure probiotic efficacy throughout shelf life in ambient or low‐moisture food systems.

## Disclosure

Parts of the research presented in this paper were previously included in Jessie Payne’s thesis titled *Evaluation of the Stability and Viability of Various Bacillus Strains as Probiotics*, completed at *Oklahoma State University* [26]. All information was cross‐checked against the original content, and the authors carefully reviewed and edited the text afterward. The final version reflects the authors’ own work and interpretation for publication.

## Conflicts of Interest

The authors declare no conflicts of interest.

## Author Contributions

Jessie Payne, Ph.D.: conceptualization, methodology, validation, formal analysis, data curation, investigation, visualization, project administration, writing—original draft, writing—review and editing. Danielle Bellmer, Ph.D.: conceptualization, methodology, validation, project administration, supervision, funding acquisition, resources, writing—review and editing. Ravi Jadeja, Ph.D.: conceptualization, supervision, funding acquisition, resources, writing—review and editing. Brooke Holt, Bailey Holcomb, and Sarah Spring: data curation, writing—review and editing.

## Funding

This research was funded by the Orville and Helen Buchanan Professorship and the National Institute of Food and Agriculture, 10.13039/100005825 (2023‐67018‐39830).

## Supporting information


**Supporting Information** Additional supporting information can be found online in the Supporting Information section. Supporting information has been submitted with this manuscript, including graphs that illustrate the effect of storage temperature on cookies and crackers containing the four probiotics tested in this study. In Figures S1 and S2, they display data for three different storage conditions: (A) freezer (−18°C), (B) refrigeration (4°C), and (C) room temperature (25°C). These graphs detail the viability of each probiotic strain over time under each temperature condition, highlighting differences in stability and survival rates. The data offer insights into how temperature influences both the shelf life and functional properties of the probiotic‐containing products, providing valuable information for optimizing storage conditions for probiotic foods.

## Data Availability

Data are available on request from the authors.
