# Supplementary material for: Storage Temperature Effects on Bacillus Spores and Lactobacillus acidophilus Viability
Source: Int J Food Sci. 2025 Sep 22;2025:3966944. doi: 10.1155/ijfo/3966944 (PMC12451811; doi:10.1155/ijfo/3966944)
Supplement: Supplementary file 1 — Supporting Information Additional supporting information can be found online in the Supporting Information section. Supporting information has been submitted with this manuscript, including graphs that illustrate the effect of storage temperature on cookies and crackers containing the four probiotics tested in this study. In Figures S1 and S2, they display data for three different storage conditions: (A) freezer (−18°C), (B) refrigeration (4°C), and (C) room temperature (25°C). These graphs detail the viability of each probiotic strain over time under each temperature condition, highlighting differences in stability and survival rates. The data offer insights into how temperature influences both the shelf life and functional properties of the probiotic‐containing products, providing valuable information for optimizing storage conditions for probiotic foods. [file IJFO-2025-3966944-s001.docx]

**Figure 1. Effect of storage temperature on cookies containing probiotics: A. Freezer (-18°C),** **B. Refrigeration (4°C), and C. Room Temperature (25°C)** **conditions.**

*Note: Different capital letters (A) at the same time point (i.e., Day 0, 1 month, etc.) denote significant differences (p < 0.05) between probiotics at that specific time point*

**Figure 2. Effect of storage temperature on crackers containing probiotics: A. Freezer (-18°C),** **B. Refrigeration (4°C), and C. Room temperature (25°C)** **conditions.**

*Note: Different capital letters (A) at the same time point (i.e., Day 0, 1 month, etc.) denote significant differences (p < 0.05) between probiotics at that specific time point*

**Table 1. The water activity of cookies during 12 months of storage: statistical values**

| **Months** | **Room temperature (25°C)** | **Refrigeration (4°C)** | **Freezer (-18°C)** |
| --- | --- | --- | --- |
| **0** | A | A | A |
| **1** | BCa | BCa | Aa |
| **2** | ABa | ABC | Aa |
| **4** | ABCa | ABCa | ABa |
| **6** | Aa | BDCb | Aab |
| **8** | ABa | ABCDb | Aab |
| **10** | ABCa | CDb | Aa |
| **12** | Cb | Dc | Aa |

*Note. Different capital letters (A) denote significant differences (p ≤ 0.05) between the same holding temperature over the storage period. Different lowercase letters (a) denote significant differences (p ≤ 0.05) between the different holding temperatures.*

**Table 2. The water activity of crackers during 12 months of storage: statistical values**

| **Months** | **Room temperature (25°C)** | **Refrigeration (4°C)** | **Freezer (-18°C)** |
| --- | --- | --- | --- |
| **0** | A | A | A |
| **1** | ABCa | Aa | Aa |
| **2** | ABCa | Aa | BCa |
| **4** | Aa | Aa | Aa |
| **6** | Ca | Ab | Aab |
| **8** | Aa | Ab | Aa |
| **10** | ABCb | Ac | Aa |
| **12** | AB | A | A |

*Note. Different capital letters (A) denote significant differences (p ≤ 0.05) between the same holding temperature over the storage period. Different lowercase letters (a) denote significant differences (p ≤ 0.05) between the different holding temperatures.*
